# Supplementary material for: Emergence failure of early epidemics: A mathematical modeling approach
Source: PLoS One. 2024 May 29;19(5):e0301415. doi: 10.1371/journal.pone.0301415 (PMC11135784; doi:10.1371/journal.pone.0301415)
Supplement: Attachment — Submitted filename: Breban.pdf [file pone.0301415.s001.pdf]

Reviewer's report on the paper *Emergence failure of early epidemics: A mathematical modeling approach*

The author consider the emergence of an epidemic through a mutation of a weak pathogen to a highly transmissible one in one or several steps for both directly transmitted and vector borne disease. As the title suggest, the author is interested in the failure of such a mutation to cause an epidemic. The starting points are the standard macroscopic deterministic models such as SIR in the directly transmitted disease and SIRSI in the vector-borne one. The possibility of the emergence of a new version of the pathogen is accounted for by splitting the infective class(es) into two (or more) classes infected by wild-type (benign) and mutant (malignant) strains of the patogen (and an analogous split of the recovered classes). The author considers the problem at the point of the disease invasion which allows him for a significant, though formal, linearization of the problem. For the SIR model with two strains, the resulting system is linear in the infectives. Such a system can lead to extinction of the infectives as $t \to \infty$. As I understand, the author is interested, however, in the extinction "after an arbitrary large time intervals" and thus he introduces a continuous time Markov chain whose expectations satisfy the above mentioned system of deterministic equations. Due to the ambiguities in the definitions, the author should precisely define what he means by the Markov chain going extinct and what is meant "after an arbitrary large time intervals"? The author's idea is to consider the so-called "patient minus one" who is infected with the wild-strain and writes down the total probability equation for the failure of this patient to start the epidemics which can occur through the infection of another patient with the wild strain and both failing, recovery of the "patient minus one" or undergoing mutation and becoming patient zero and subsequently failing. This leads to a functional dependence between the probability p_1 of the failure of patient zero and the probability p_1' of the patient minus one's failure. It is interesting that the earlier paper of Kendall, of which this paper is a generalization, there is a close relation between the stability of the disease free equilibrium of the deterministic system and the probability of failure, that is, the probability of failure is the reciprocal of the basic reproduction number if the latter is greater than one and, obviously, it is equal to one otherwise. The authors fails to mention whether there is such a relation here. In principle, the emergence of the disease can be modelled as the establishment of an endemic (non-zero) equilibrium of I. Is there any connection with the asymptotic stability of the derived deterministic system of equations in this paper?

This argument is repeated for two other models. First, in the SIR model the author allows for the pathogen to undergo several mutations. Following the construction of the Markov process from the first part, the author derives the probability of the extinction of the virulent mutation as a composition of the functional dependence derived above. The main result here is to consider the situation where mutations between different strains have the same properties in which case the probability of extinction after n mutations is a solution of a discrete dynamical system with the initial condition being the probability of the

epidemic extinction started by patient zero. The author then investigates the stability of the fixed point of this system. Again, seems to be a close relation between the obtained results and the parameters of the deterministic system which would be interesting to elaborate on.

Summarizing, I found the paper interesting and well-written. Nevertheless, as probabilistic models are not my strong point, I would appreciate if the link with the dynamics of the original deterministic models was better explained.
