# Supplementary material for: Emergence failure of early epidemics: A mathematical modeling approach
Source: PLoS One. 2024 May 29;19(5):e0301415. doi: 10.1371/journal.pone.0301415 (PMC11135784; doi:10.1371/journal.pone.0301415)
Supplement: Attachment — Submitted filename: Rebuttal_.docx [file pone.0301415.s003.docx]

**ANSWERS TO THE REVIEWERS**

**REVIEWER 1**

We retyped the comments and suggestions of the referee in italic face and broke the text into points, which we answered immediately in blue font:

*Reviewer’s report on the paper Emergence failure of early epidemics: A mathematical modeling approach*

*The author consider the emergence of an epidemic through a mutation of a weak pathogen to a highly transmissible one in one or several steps for both directly transmitted and vector borne disease. As the title suggest, the author is interested in the failure of such a mutation to cause an epidemic. The starting points are the standard macroscopic deterministic models such as SIR in the directly transmitted disease and SIRSI in the vector-borne one. The possibility of the emergence of a new version of the pathogen is accounted for by splitting the infective class(es) into two (or more) classes infected by wild-type (benign) and mutant (malignant) strains of the patogen (and an analogous split of the recovered classes). The author considers the problem at the point of the disease invasion which allows him for a significant, though formal, linearization of the problem. For the SIR model with two strains, the resulting system is linear in the infectives. Such a system can lead to extinction of the infectives as t → ∞. As I understand, the author is interested, however, in the extinction ”after an arbitrary large time intervals” and thus he introduces a continuous time Markov chain whose expectations satisfy the above mentioned system of deterministic equations.*

*Due to the ambiguities in the definitions, the author should precisely define what he means by the Markov chain going extinct and what is meant ”after an arbitrary large time intervals”?*

>>> We introduced definitions for the extinction times of the Markov chains presented in Tables 1 and 2.

>>> The extinction of the Markov chain in Table 1 is addressed on pages 4 and 5, lines 119-124 in the revised manuscript: “Markov chains can be naturally used to model population extinction. For the Markov chain defined in Table 1, the time to extinction is given by inf{t ≥ 0 : i(t) = 0, i′(t) = 0}, where i(t) and i′(t) depend on the model parameters and initial condition. The extinction probability is the fraction of times the Markov chain trajectories go extinct. However, to obtain the probability that patient minus one fails to initiate an epidemic, we do not invoke the definition.”

>>> The extinction of the Markov chain in Table 2 is addressed on page 10, lines 271-274 in the revised manuscript: “The extinction time of this Markov chain is defined as inf{t ≥ 0 : ih(t) = 0, ih(t) = 0, i′h(t) = 0, i′v(t) = 0}, where the stochastic variables depend on the model parameters and initial condition. The extinction probability is given by the fraction of times the Markov chain trajectories go extinct.”

*The author’s idea is to consider the so-called ”patient minus one” who is infected with the wild-strain and writes down the total probability equation for the failure of this patient to start the epidemics which can occur through the infection of another patient with the wild strain and both failing, recovery of the ”patient minus one” or undergoing mutation and becoming patient zero and subsequently failing. This leads to a functional dependence between the probability p1 of the failure of patient zero and the probability p′1 of the patient minus one’s failure.*

>>> We agree with the reviewer about these points. The phrase “patient minus one” is only meant to suggest a disease case occurring before patient zero.

*It is interesting that the earlier paper of Kendall, of which this paper is a generalization, there is a close relation between the stability of the disease free equilibrium of the deterministic system and the probability of failure, that is, the probability of failure is the reciprocal of the basic reproduction number if the latter is greater than one and, obviously, it is equal to one otherwise. The authors fails to mention whether there is such a relation here. In principle, the emergence of the disease can be modelled as the establishment of an endemic (non-zero) equilibrium of I. Is there any connection with the asymptotic stability of the derived deterministic system of equations in this paper?*

>>> The reviewer has a very pertinent question, which I would rephrase more simply as: Is it possible to relate p1 and/or p’1 to dynamical elements of the original ODE model? I do not have an answer to this question, but I propose three points of discussion below.

>>> **The simplicity of the Kendall process can be deceiving**. The Kendall process is a simple, one-dimensional Markov chain for the number of infectious individuals, with two parameters β and γ. The expected number of infected individuals satisfies dI/dt=βI-γI. Choosing the time unit such that γ=1, we find that, in fact, the Kendall process has just one parameter, β/γ=R0. Simple considerations lead to the fact that p1 must be decreasing from 1 as a function of R0. All these result without an actual analysis of the Markov chain.

>>> **p’1 needs more parameters than those determining the stability of the ODE dynamics.** When Markov chains are more complex than the Kendall process, they play a larger role in determining p1 or p’1. For the Markov chain in Table 1, we obtained that p’1 is a function of three parameters: R’0, m’ and R0. (Note that the same parameterization is used by Antia et al. 2003.) Therefore, p’1 depends on other parameters than those governing the stability of the dynamics; i.e., m’. Furthermore, it is important to note that p’1 does not result as a unique function associated with the Markov chain. Rather, there exist many Markov chains sharing the same formulae for p’1. In our discussion on pages 5 and 6 of the revised manuscript, we chose β and β’ at our convenience; all other Markov chain parameters were determined by this choice and the parameters R’0, m’ and R0.

>>> The last point to make is that our results are limited to just a few Markov chains. Therefore, it is difficult to infer general statements about how p’1 may relate to the ODE dynamics.

*This argument is repeated for two other models. First, in the SIR model the author allows for the pathogen to undergo several mutations. Following the construction of the Markov process from the first part, the author derives the probability of the extinction of the virulent mutation as a composition of the functional dependence derived above. The main result here is to consider the situation where mutations between different strains have the same properties in which case the probability of extinction after n mutations is a solution of a discrete dynamical system with the initial condition being the probability of the epidemic extinction started by patient zero. The author then investigates the stability of the fixed point of this system. Again, seems to be a close relation between the obtained results and the parameters of the deterministic system which would be interesting to elaborate on.*

*Summarizing, I found the paper interesting and well-written. Nevertheless, as probabilistic models are not my strong point, I would appreciate if the link with the dynamics of the original deterministic models was better explained.*

>>> We thank the reviewer for acknowledging our work.

>>> It is difficult to establish how p’1 relates to the ODE dynamics. This remains for further work.

**REVIEWER 2**

We retyped in italics the comments and suggestions of the referee that were not directly answered by the proposed changes in the main text. We answered them immediately using blue font.

Top of page 1: “Very nice approach and results. The writing and presentation needs some work, as do the figures.”

>>> We thank the reviewer for acknowledging our work. We made numerous changes, throughout the manuscript in reply to the comments and suggestions of two anonymous reviewers.

Page 2, third comment from the top: “Better to just pick one.“

>>> We chose “he” over “she”.

Page 5: “How can it be both? Explain.”

>>> We simply rephased it as R0’.

Page 6: “Need a better word. “from”?”

>>> We rephrased as: “the singularity in the denominator does not occur in Eq. (9) ”

Page 8, line 246: The reviewer proposes “are satisfied numerically”.

>>> Instead, we used “are verified numerically”.

Page 10, line 300: “Where are these defined?”

>>> p10 and p01 result from Eq (42), just above, that introduces pjk for all natural numbers j and k.

Page 14, line 391: “Was SARS really a pandemic?”

>>> We replaced “cause a pandemic” with “spread worldwide”.
