# Supplementary material for: Emergence failure of early epidemics: A mathematical modeling approach
Source: PLoS One. 2024 May 29;19(5):e0301415. doi: 10.1371/journal.pone.0301415 (PMC11135784; doi:10.1371/journal.pone.0301415)
Supplement: Attachment — Submitted filename: Rebuttal_.docx [file pone.0301415.s005.docx]

**ANSWERS TO THE REVIEWERS**

**REVIEWER 1**

We retyped the comments and suggestions of the referee in italic face and broke the text into points, which we answered immediately in blue font:

*My all concerns have been appropriately addressed and the paper can be published in its current form.*

>>> We thank the reviewer for his/her positive remark.

**REVIEWER 2**

We retyped in italics the comments and suggestions of the referee that were not directly answered by the proposed changes in the main text. We answered them immediately using blue font.

*Reviewer #2: See attached marked-up manuscript.*

>>> We made all changes suggested by the reviewer.
